# Tibetan *Fritillaria cirrhosa* D. Don Extract Ameliorates DSS-Induced Ulcerative Colitis by Repairing Damage to the Intestinal Mucosal Barrier and Regulating the Gut Microbiota

**DOI:** 10.3390/nu18060970

**Published:** 2026-03-19

**Authors:** Zhengyang Hao, Xiangjun Chen, Qing Peng, Ruipeng Wu, Huan Zhang, Ping Yin, Xuanfu Yu, Shaokang Wang

**Affiliations:** 1 Clinical Medical Research Center for Plateau Gastroenterological Disease of Xizang Autonomous Region, School of Medicine, Xizang Minzu University, Xianyang 712082, China; haozy6@163.com (Z.H.); xjchen@xzmu.edu.cn (X.C.); m18956456664@163.com (Q.P.); wurp@xzmu.edu.cn (R.W.); pingyin0106@163.com (P.Y.); 18717606046@163.com (X.Y.); 2 Key Laboratory of Environmental Medicine and Engineering of Ministry of Education, Department of Nutrition and Food Hygiene, School of Public Health, Southeast University, Nanjing 210009, China; huanzhang2023@163.com

**Keywords:** Tibetan *Fritillaria cirrhosa* D. Don extract, ulcerative colitis, mucosal barrier, gut microbiota

## Abstract

**Background/Objectives:** Ulcerative Colitis (UC) is a chronic inflammatory disease of the colon that profoundly impacts human health. Conventional pharmacological treatments are associated with serious adverse reactions and toxic side effects. Consequently, the development of natural plant-derived biological agents for UC treatment is an urgent imperative. **Methods:** Utilising a Dextran Sulfate Sodium (DSS)-induced ulcerative colitis mouse model, with mice receiving low, medium, and high doses of water extract of Tibetan *Fritillaria cirrhosa* D. Don extract (FCD), alongside a group receiving 5-aminosalicylic acid. The Disease Activity Index (DAI) was calculated, colon length was measured, histological scores were assessed, and histopathological alterations were evaluated. Inflammatory factor were determined by ELISA; mRNA and protein expression in colonic tissue was analysed by RT-qPCR and Western blotting; intestinal barrier-related proteins were examined by immunofluorescence and immunohistochemistry; and gut microbiota composition was assessed by 16S rRNA sequencing. **Results:** Research has confirmed that FCD alleviates symptoms of DSS-induced colitis in mice, specifically manifested by a slower rate of weight loss, reduced colon shortening, and decreased disease activity index. It has been demonstrated that the process under investigation exerts a beneficial effect on intestinal injury by means of a number of mechanisms. These include increased goblet-cell production, elevated IL-10 levels, and reduced levels of TNF-α, IL-1β, and IL-6. Furthermore, immunofluorescence detection, immunohistochemical analysis, and RT-qPCR results indicate that FCD maintains the integrity of the intestinal mucosal barrier by enhancing the expression of Zonula occludens-1 (ZO-1), occludin, and claudin-1 proteins and their corresponding mRNAs, in addition, FCD can regulate the gut microbiota and promote its diversity. **Conclusions:** Research indicates that FCD may exert therapeutic effects on ulcerative colitis (UC) by regulating intestinal barrier integrity and modulating the gut microbiota. These findings reinforce the idea that FCD could be used as a natural therapy to improve UC.

## 1. Introduction

Ulcerative Colitis (UC) is classified as a chronic inflammatory bowel disease (IBD) that primarily affects the rectum, colonic mucosa, and submucosal layer [1]. The symptoms primarily manifest as weight loss, diarrhoea with blood in the stool, and immune dysregulation [2,3]. UC is characterised by recurrent episodes, a protracted course, and a complex, multifactorial pathogenesis, with its precise mechanisms remaining unclear [4,5]. The incidence of the disease is rising in newly industrialised nations while remaining high in Western developed countries. In 2023, the global prevalence of ulcerative colitis is estimated to reach a maximum of 5 million cases, with its incidence continuing to rise on a worldwide basis [6,7]. Several factors have been posited as potential triggers for UC onset, including disruption of the gut microbiota, metabolic disorders, damage to the intestinal barrier, and alterations in intestinal immune regulation [8]. Current treatment modalities for UC principally comprise drug therapy, surgical interventions, and the use of biological inhibitors [9,10]. However, conventional drug treatments have shown limited efficacy, and prolonged use may lead to adverse effects. The primary function of these medications is to reduce inflammation.

The intestinal barrier comprises epithelial cells, a mucus layer, and tight junction proteins. Enhancing intestinal barrier function may exert a protective effect against UC [11]. Impaired intestinal barrier function is commonly associated with various gastrointestinal disorders. Down-regulation of tight junction (TJ) protein expression in intestinal epithelial cells has been demonstrated to increase intestinal permeability, allowing harmful substances such as bacteria and toxins to enter the bloodstream. Experimental studies have shown that UC patients who achieve mucosal healing exhibit superior long-term clinical remission outcomes compared with those who fail to attain mucosal healing [12]. Moreover, the gut microbiota plays a pivotal role in the development of inflammatory bowel diseases [13,14,15,16,17]. *Chinese yam (Dioscorea) polysaccharide*, Tetrastigma hemsleyanum polysaccharide, Hypecoum leptocarpum and multiple natural products can mediate anti-inflammatory and mucosal repair effects by regulating microbial composition and activity [18,19,20,21]. Consequently, preserving the intestinal barrier and regulating the gut microbiota are recognised as pivotal objectives in the prevention and treatment of UC. At present, 5-aminosalicylic acid (5-ASA) is used in the management of UC. This synthetic compound is a member of the nonsteroidal anti-inflammatory drug (NSAID) class, primarily acting upon the intestinal mucosa to inhibit prostaglandin synthesis, scavenge oxygen free radicals, and reduce intestinal permeability. However, its use may cause adverse effects, including abdominal discomfort, nausea, weight loss, diarrhoea, and headaches [5,22,23]. Therefore, identifying safe and effective treatment options has become a priority for current research.

The genus *Fritillaria* is one of the most extensive genera within the family Liliaceae [24]. It is native to several Asian countries, including China, Korea, and Japan. FCD is a plateau-endemic medicinal plant belonging to the *Fritillaria* genus in the lily family [25]. It plays a distinctive role in treating digestive-system ailments in Tibetan medical tradition. Primarily cultivated on the Qinghai–Tibet Plateau at altitudes of 3000 and 4500 m and serves as a key medicinal herb for addressing gastrointestinal heat syndromes. Tibetan medical theory holds that the bitter and cooling properties of the herb clear intense heat from the stomach and intestines, detoxify the body, and promote wound healing. Recent studies have indicated that FCD exerts a mitigating effect on the development of non-alcoholic fatty liver fibrosis in murine models [26]. However, in-depth research into the pharmacological mechanisms of FCD’s effects on the gastrointestinal tract remains largely unexplored.

This study aimed to investigate the efficacy of FCD extract in treating Dextran Sulfate Sodium (DSS)-induced UC in mouse. This pioneering work represents the first demonstration that FCD effectively mitigates colonic tissue damage and inflammatory responses in a DSS-induced UC model by regulating intestinal barrier integrity and maintaining gut microbiota homeostasis. These findings provide novel insights for developing platform-based gastrointestinal protective therapeutics.

## 2. Materials and Methods

### 2.1. Reagents and Materials

FCD was procured from the Tibet Snow Region Pharmacy Chain Co., Ltd. (Lhasa, China). DSS and 5-ASA were obtained from SAITONG (Tianjin, China). The enzyme-linked immunosorbent assay (ELISA) kits for IL-6, IL-10, IL-1β, and TNF-α were procured from Enzyme Immunoassay (Shanghai, China). The protein quantification kit for determining protein concentration was purchased from Servicebio (Wuhan, China). The following antibodies were purchased from Affinity: Zonula occludens-1 (ZO-1), claudin-1, occludin, Glyceraldehyde-3-Phosphate Dehydrogenase (GAPDH) and β-Tubulin.

### 2.2. Preparation of FCD Extract

A quantity of 100 g of FCD should be added to 1 L of ultrapure water, and the mixture then subjected to the process of decoction for a period of three hours. The decoction should then be collected, after which a further 500 mL of ultrapure water should be added and the mixture should be left to decoct for a period of 1.5 h. The decoctions should then be collected and combined. The decoction should then be concentrated by rotary evaporation, after which the process of drying should be undertaken using a vacuum freeze-dryer (Qingdao Yonghe Chuangxin Electronic Technology Co., Ltd., Qingdao, China). Prior to use, the lyophilized FCD extract powder must be stored at −80 °C.

### 2.3. UPLC-MS/MS Analysis of FCD

For the analysis of FCD, This project utilises an ultra-high performance liquid chromatography system (Vanquish, Thermo Fisher Scientific, Waltham, MA, USA) for the analysis of FCD. The chromatographic separation of the target compounds was achieved using a Pheomenex Kinetex C18 column (2.1 mm × 100 mm, 2.6 µm, Torrance, CA, USA). Liquid Chromatography (LC) mobile phase A was aqueous, containing 0.01% acetic acid, while phase B comprised a mixture of isopropanol and acetonitrile at a volume ratio of 1:1. The temperature of the sample tray is indicated as follows: 4 °C. Sample volume is 2 μL.

### 2.4. Animals

The experiment was conducted using male C57BL/6 mice, which were obtained from the China Dashuo Animal Experiment Centre and were of a clean-grade and healthy variety. The subject is male, aged between 6 and 8 weeks, with a weight range of 20–22 g. The animal use licence number is as follows: SCXK(Chuan)2025-0030. The mice were housed at the Experimental Animal Centre of Tibet University for Nationalities under the following conditions: maintain a temperature of (25 ± 2) °C and an air humidity of (50 ± 2)%, with a 12 h light-dark cycle, and provide ample food and water.

### 2.5. Experimental Design

Forty-eight mice were randomly divided into six groups, with eight mice in each group, a control group, a DSS group, a positive drug group (5-ASA), a low-dose FCD group (FCD-L), a medium-dose FCD group (FCD-M), and a high-dose FCD group (FCD-H). During the experimental period, in the preceding three days, the Control, DSS, and 5-ASA groups were administered normal water via gavage. The FCD-L, FCD-M and FCD-H groups were administered FCD extract concentrations of 225 mg/kg, 450 mg/kg and 900 mg/kg, respectively, via gavage. From the fourth day onwards, with the exception of the control group, the remaining groups were provided with drinking water that had been supplemented with 3% sodium dextran sulphate, with the objective of inducing UC. Concurrently, the 5-ASA group received 100 mg/kg 5-aminosalicylic acid via gavage, while the FCD-L, FCD-M, and FCD-H groups continued gavage at the aforementioned concentrations. On the tenth day, the mice were euthanised by cervical dislocation [27]. Collect serum, colon, liver, spleen, and caecal contents.

### 2.6. Disease Activity Index Analysis and Histopathology

A daily assessment of the DAI is to be conducted following the administration of the DSS treatment [28,29]. Subsequent to the conclusion of the experiment, photographs were taken of the anus, colon, and spleen, and measurements were taken of the length of the colon from the caecum to the anus and of the spleen. For the purpose of histopathological assessment, fix colon and liver tissue sections with 4% paraformaldehyde. Paraffin-embedded colonic specimens were sectioned for haematoxylin and eosin (H&E) staining and Alcian Blue—Periodic Acid Schiff (AB-PAS) staining. The 3D digital slide scanner model P250 FLASH (Dangier, Jinan, China) was utilised in this study.

### 2.7. Alcian Blue—Periodic Acid Schiff Stain Staining

Following deparaffinization and rehydration, colonic tissue sections from mice were subjected to Alcian blue staining (for acidic mucins) and the periodic acid-Schiff reaction (for neutral mucins). The sections were then counterstained with hematoxylin to visualise nuclei, followed by dehydration, clearing, and mounting. This staining protocol was employed to distinguish goblet cells and characterise mucin types.

### 2.8. Cytokine Assay

The levels of cytokines IL-1β, IL-6, IL-10, and TNF-α in serum samples were measured using an enzyme-linked immunosorbent assay (ELISA) kit, with the experimental methodology adhering to the kit instructions.

### 2.9. Immunofluorescence and Immunohistochemistry

For the purpose of immunofluorescence analysis, colon sections were deparaffinised and rehydrated prior to being subjected to an overnight incubation at 4 °C with antibodies ZO-1 (1:100) and occludin (1:200). The sections were then subjected to incubation with CY3H- and FITC-labelled secondary antibodies, with nuclei counterstained using DAPI. Image acquisition was performed using a 3D digital slide scanner, and the mean fluorescence intensity per image was determined using ImageJ software (V1.52a) analysis system.

For the purpose of immunohistochemical analysis, colon tissue sections underwent a process of dewaxing and rehydration, employing a combination of xylene and graded ethanol solutions. Antigen retrieval was subsequently performed by heating the sections in boiling sodium citrate buffer within an induction furnace. The primary antibody, ZO-1, was applied at a concentration of 1 mg/mL for a period of 12 h at a temperature of 4 °C. Subsequently, horseradish peroxidase-labelled secondary antibody was applied, followed by 3,3′-Diaminobenzidine (DAB) staining, haematoxylin counterstaining, counterstaining with blue in running water, dehydration with graded ethanol solutions and xylene, and coverslipping. A semi-quantitative analysis of the target protein was performed using ImageJ software, with protein expression intensity represented by the area occupied by positive protein.

### 2.10. Western Blot Analysis

The extraction of total colonic proteins was then undertaken using Radioimmunoprecipitation Assay Lysis Buffer. The centrifuge should be operated at 12,000 rpm for a period of 15 min. Proteins were subsequently extracted, and their concentrations were determined using a Bicinchoninic Acid Assay (BCA) protein assay kit based on the biuret reaction. The primary antibodies employed in this study were: GAPDH, β-Tubulin, ZO-1, occludin, and claudin-1. Subsequently, secondary antibodies were incubated at room temperature for 1 h, and protein bands were captured using an automated chemiluminescent imaging system. The relative expression of target proteins was then normalised against corresponding GAPDH and β-Tubulin internal controls.

### 2.11. Reverse Transcription Quantitative Polymerase Chain Reaction

Total RNA was extracted from colon tissue using the M5 Universal RNA Mini Kit (Mei5bio, Beijing, China), with RNA concentration and purity determined using an NanoDrop Microvolume Spectrophotometers (Thermo Scientific, Waltham, MA, USA). Utilising GAPDH as the housekeeping gene, the relative expression levels of target genes were calculated by means of the 2^−ΔΔCt^ method. The primer sequences for RT-qPCR are listed in Table 1. The RT-qPCR primer sequences are listed in Table 1. Primers were purchased from Shanghai Sanang Biotechnology Co., Ltd. (Shanghai, China).

### 2.12. 16SrRNA Sequencing

Intestinal content samples were dispatched to Novogene Co., Ltd. (Beijing, China). The 16S rRNA gene sequencing was conducted in Beijing, China. The workflow encompassed a series of meticulous procedures, including DNA extraction, PCR amplification, magnetic bead purification and recovery, quantitative fluorescence analysis, library preparation, and high-throughput sequencing. The primers 515F (5′-GTGCCAGCMGCCGCGGTAA-3′) and 806R (5′-GGACTACHVGGGTWTCTAAT-3′) were used to target the V3–V4 hypervariable region of the bacterial 16S rRNA gene. After quantifying the purified PCR products using Qubit and qPCR, sequencing was performed on either the NovaSeq 6000 (Illumina Inc., San Diego, CA, USA), including principal coordinate analysis (PCoA), non-metric multidimensional scaling (NMDS), α diversity analysis, and bacterial abundance difference analysis were all performed using Novigen’s cloud platform (www.novogene.com, accessed on 6 October 2025).

### 2.13. Statistical Analysis

Data analysis and bar chart creation were performed using GraphPad Prism 10.1.2 software. Statistical differences were determined using one-way analysis of variance (ANOVA) combined with Tukey’s post hoc test, with significance levels set at *p* < 0.05.

## 3. Results

### 3.1. Identification of FCD Extract

**Figure 1:**
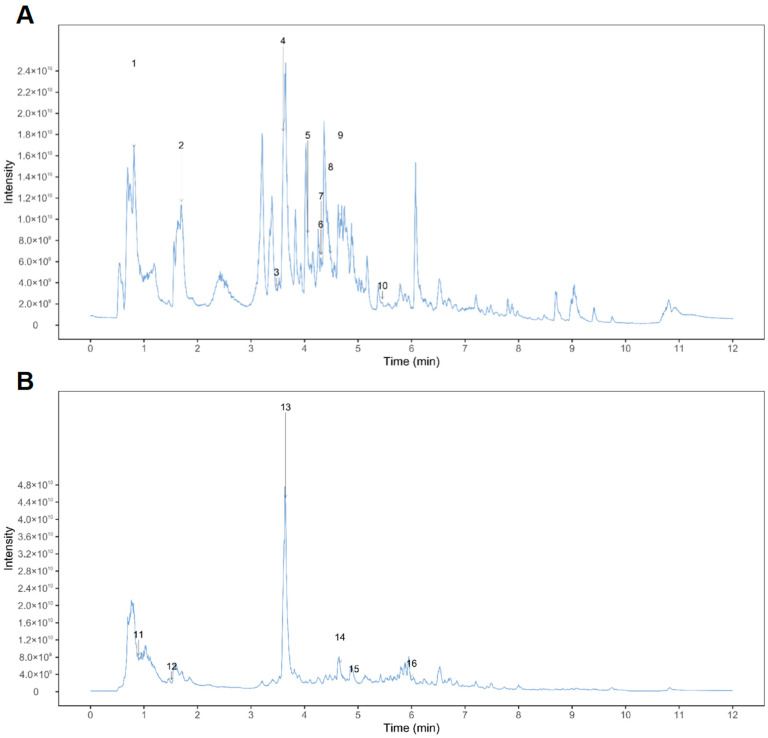
Total ion chromatograms of FCD extracts analysed by UPLC-MS/MS. (**A**) UPLC-MS/MS analysed Positive ion mode; (**B**) UPLC-MS/MS analysed Negative ion mode. The corresponding numbers correspond to the order in Table 2.

The major components of FCD extract were determined by Mass spectrometry/mass spectrometry chromatography (Figure 1A,B). The components of the identified compounds are shown in Table 2.

### 3.2. FCD Alleviates Symptoms of DSS-Induced Colitis in Mice

**Figure 2:**
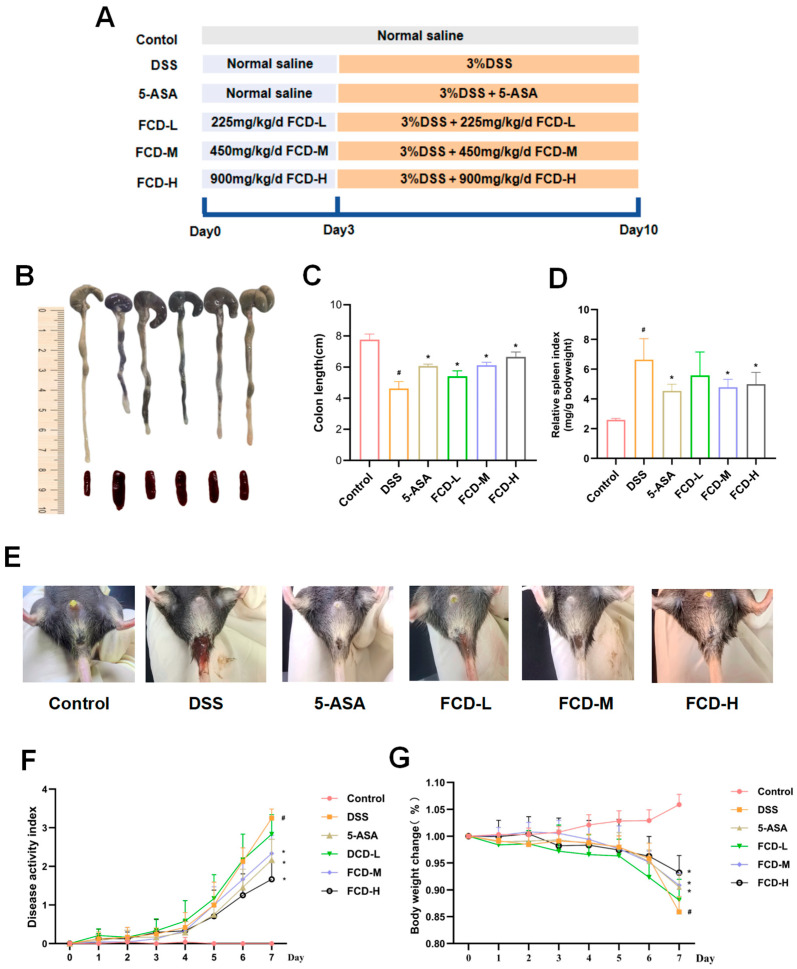
FCD alleviates DSS-induced UC. (**A**) Schematic diagram of animal grouping and administration; (**B**) Photographs of colon and spleen from different groups; (**C**) Colon length measurements; (**D**) Spleen indices; (**E**) Photographs of the anus; (**F**) DAI scoring results; (**G**) Changes in body weight. All date are presented by the mean ± SEM (*n* = 8). Compared with the CON group, # *p* < 0.05; compared with the DSS group, * *p* < 0.05.

We first evaluated the effects of FCD on UC mice (Figure 1). The results showed that compared with the control group, mice in the DSS group exhibited a significant decrease in body weight. When compared with the DSS group, all 5-ASA and FCD groups demonstrated an increase in body weight. Furthermore, the FCD-L, FCD-M, and FCD-H groups demonstrated a trend towards increased body weight (Figure 2G). Following DSS-induced colitis, DAI scores significantly increased, indicating heightened inflammatory severity in mice. However, administration of 5-ASA and FCD solution markedly reduced DAI scores (Figure 2F). Furthermore, the colon length in the DSS group was significantly shorter than that in the control group. and spleen weight ratio was significantly elevated, these symptoms were markedly alleviated in the 5-ASA and FCD treatment groups (Figure 2B–D). Observation of the mice’s anuses revealed that those in the DSS group exhibited more severe haematochezia, a symptom that improved following administration of 5-ASA and FCD (Figure 2E).

### 3.3. FCD Ameliorates Histopathological Alterations in DSS-Induced Mouse Colitis

**Figure 3:**
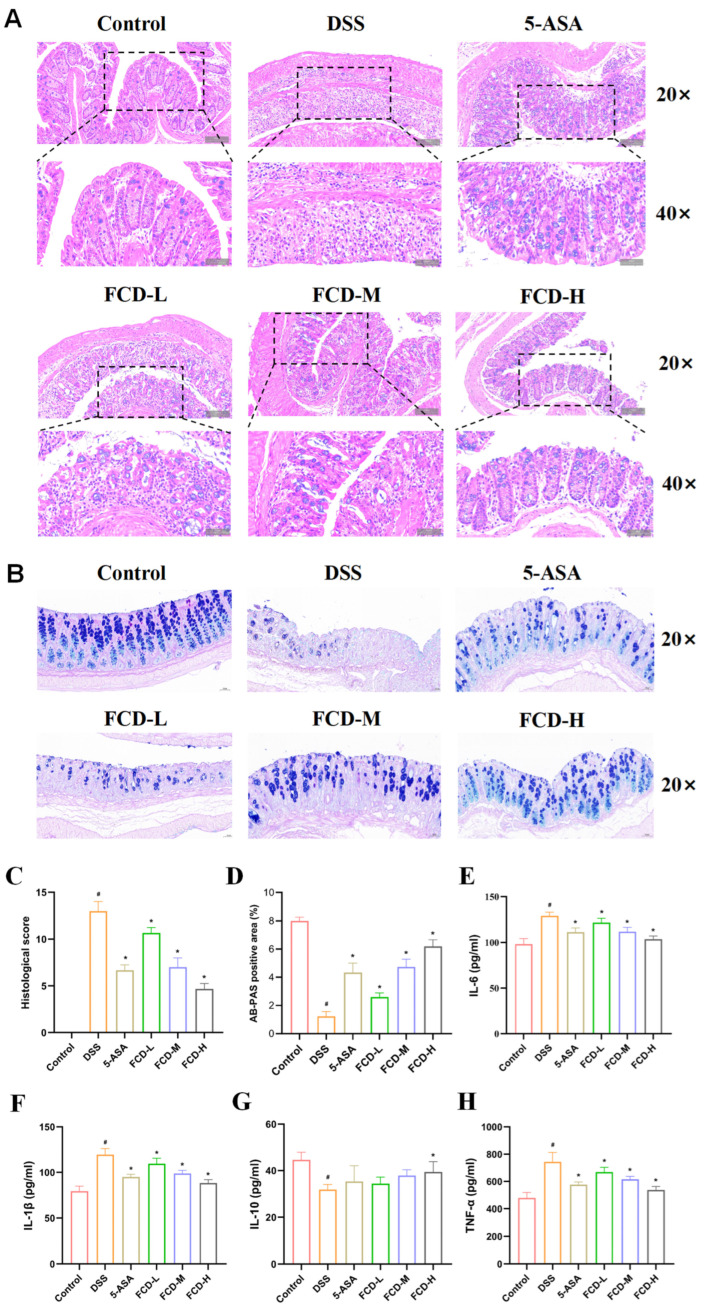
FCD protects colonic integrity and reduces inflammatory response. (**A**) H&E staining of colonic tissue (Scale bars: 100 µm and 50 µm) (*n* = 3), The black square shows a section captured under a 20× microscope corresponding to its 40× magnification. (**B**) AB-PAS staining of colonic tissue (Scale bars: 50 µm) (*n* = 3). (**C**) Histopathological scoring results for H&E-stained colonic tissue. (**D**) Area of AB-PAS-positive regions. Concentrations of IL-6 (**E**), IL-1β (**F**), IL-10 (**G**), and TNF-α (**H**) in the serum (*n* = 6). All date are presented by the mean ± SEM. Compared with the CON group, # *p* < 0.05; compared with the DSS group, * *p* < 0.05.

In H&E-stained colonic sections, the control group exhibited intact colonic architecture with no histological alterations, whereas sections from the DSS group demonstrated inflammatory cell infiltration, ulceration, crypt and marked epithelial damage (Figure 3A). Furthermore, histopathological scores in mice from the 5-ASA group and all FCD groups were lower than those in the DSS group, with a concentration-dependent reduction observed across all FCD groups (Figure 3C). Histological evaluation revealed that 5-ASA, FCD-M, and FCD-H significantly enhanced intestinal architecture and mitigated DSS-induced colonic inflammation. Liver sections revealed no significant differences across all groups (Supplementary Figure S1). The findings suggest that FCD is efficacious in ameliorating UC in murine models without causing hepatic damage.

AB-PAS-stained colonic tissue revealed a reduction in goblet cell numbers within the DSS group compared to the control group, demonstrating that intestinal mucosal damage following DSS treatment leads to diminished goblet cell populations. Both the 5-ASA and FCD groups exhibited mitigation of DSS-induced goblet cell. These findings confirm that FCD exerts a certain ameliorative effect on DSS-induced colitis in mice (Figure 2B,D).

### 3.4. Regulation of DSS-Induced Inflammatory Cytokine Levels in Mice by FCD

In order to gain further insight into the inflammatory status of FCD-treated DSS mice, serum levels of inflammatory cytokines were measured. In comparison with the control group, the mean levels of IL-6, IL-1β, and TNF-α were significantly elevated in the DSS group. In comparison with the DSS group, both 5-ASA and FCD groups demonstrated significant decreases. Concurrently, the mean level of IL-10 was found to be significantly reduced in the DSS group in comparison with the control group. Compared with the DSS group results, only the FCD-H group showed significant growth (Figure 3E–G).

### 3.5. FCD Ameliorates DSS-Induced Intestinal Barrier Damage in Mice

**Figure 4:**
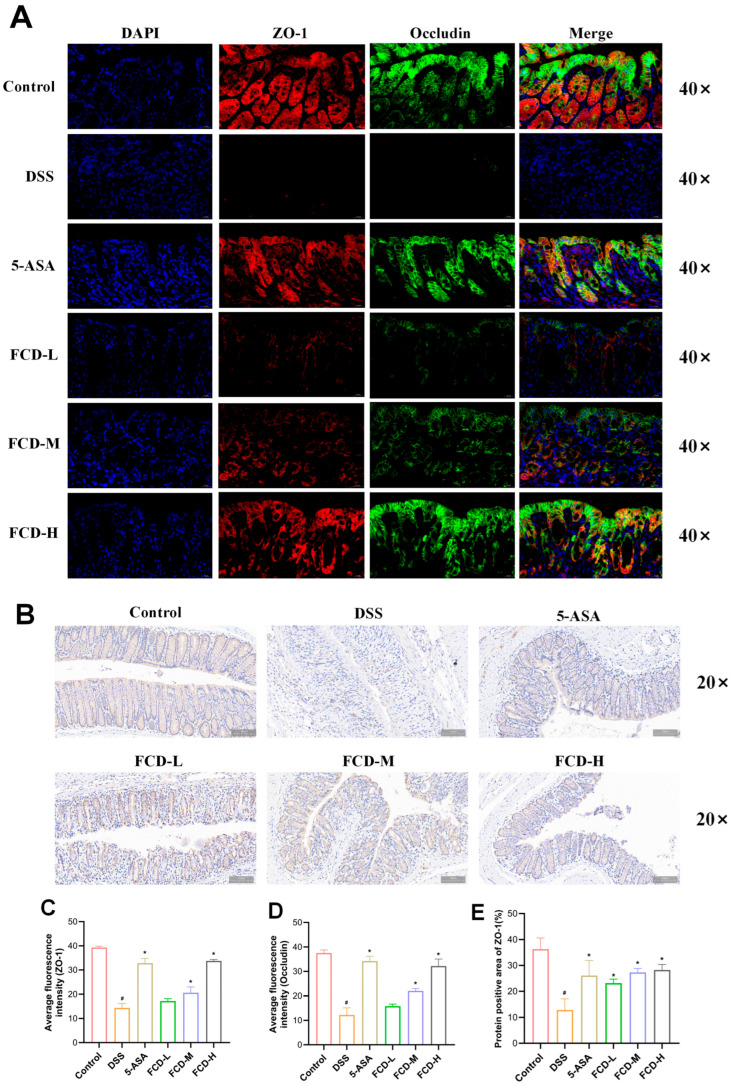
FCD improves intestinal barrier integrity in DSS-induced ulcerative colitis. (**A**) Immunofluorescence image of ZO-1 and occludin (Scale bars: 20 µm) (*n* = 3). (**B**) Immuno-histochemical analysis of ZO-1 in colonic tissues (Scale bars: 100 µm) (*n* = 3). (**C**) Fluorescence quantitative results of ZO-1. (**D**) Fluorescence quantitative results of occludin. (**E**) ZO-1 immunohistochemically positive protein areas. Compared with the CON group, # *p* < 0.05; compared with the DSS group, * *p* < 0.05.

**Figure 5:**
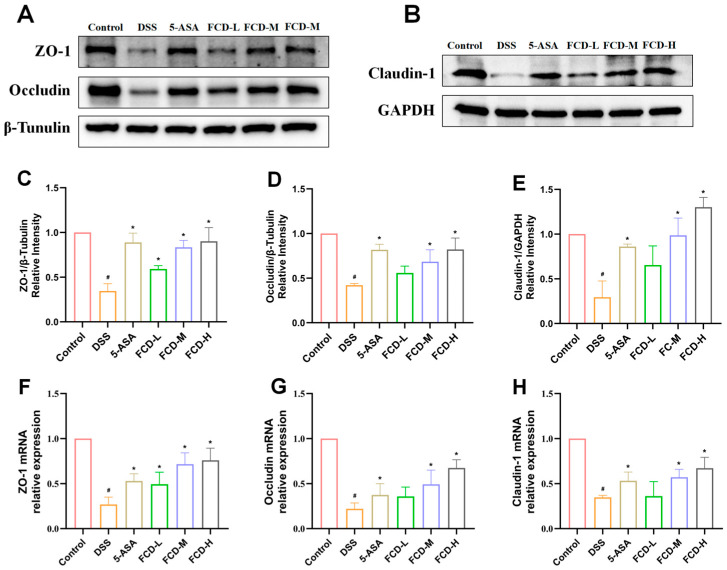
Effects of FCD on intestinal barrier function in DSS-induced colitis. (**A**) Representative expressions determined using Western blot analysis for ZO-1 and occludin (*n* = 3), β-Tunulin was used as the protein loading control. (**B**) Representative expressions determined using Western blot analysis for claudin-1 (*n* = 3), GAPDH was used as the protein loading control. (**C**–**E**) Density measurement of ZO-1 expression. Density measurement of occludin expression. Density measurement of claudin-1 expression. (**F**–**H**) The mRNA expression of ZO-1 occludin claudin-1 in colon tissue of DSS-induced UC mice was analysed using real-time qPCR (*n* = 6). All date are presented by the mean ± SEM. Compared with the CON group, # *p* < 0.05; compared with the DSS group, * *p* < 0.05.

Immunofluorescence results revealed a significant decrease in the number of ZO-1 and occludin-positive cells in the DSS group compared with the control group. In comparison with the DSS group, the 5-ASA, FCD-M, and FCD-H groups demonstrated a substantial upregulation of ZO-1 and occludin-positive cells (Figure 4A,C,D). Immunohistochemical analysis revealed that the ZO-1 protein expression levels in the DSS group were lower than those in the Control group. In comparison with the DSS group, ZO-1 expression levels increased in all 5-ASA and FCD groups, with the most pronounced increase observed in the FCD-H group. Furthermore, FCD groups exhibited a dose-dependent increase in expression (Figure 4B,E). Western blot analysis revealed that the expression levels of ZO-1, occludin, and claudin-1 were reduced in the DSS group. Conversely, there was a significant elevation in the expression levels of tight junction proteins in the 5-ASA, FCD-M, and FCD-H groups (Figure 5A–E). RT-qPCR results demonstrated that tight junction protein expression was significantly reduced in the colon of the model group, whereas it was markedly upregulated in the 5-ASA, FCD-M, and FCD-H groups, with increased transcriptional levels (Figure 5F–H). In conclusion, these findings suggest that FCD has a protective effect against DSS-induced intestinal barrier damage.

### 3.6. FCD Modulates DSS-Induced Gut Microbiota Dysbiosis

**Figure 6:**
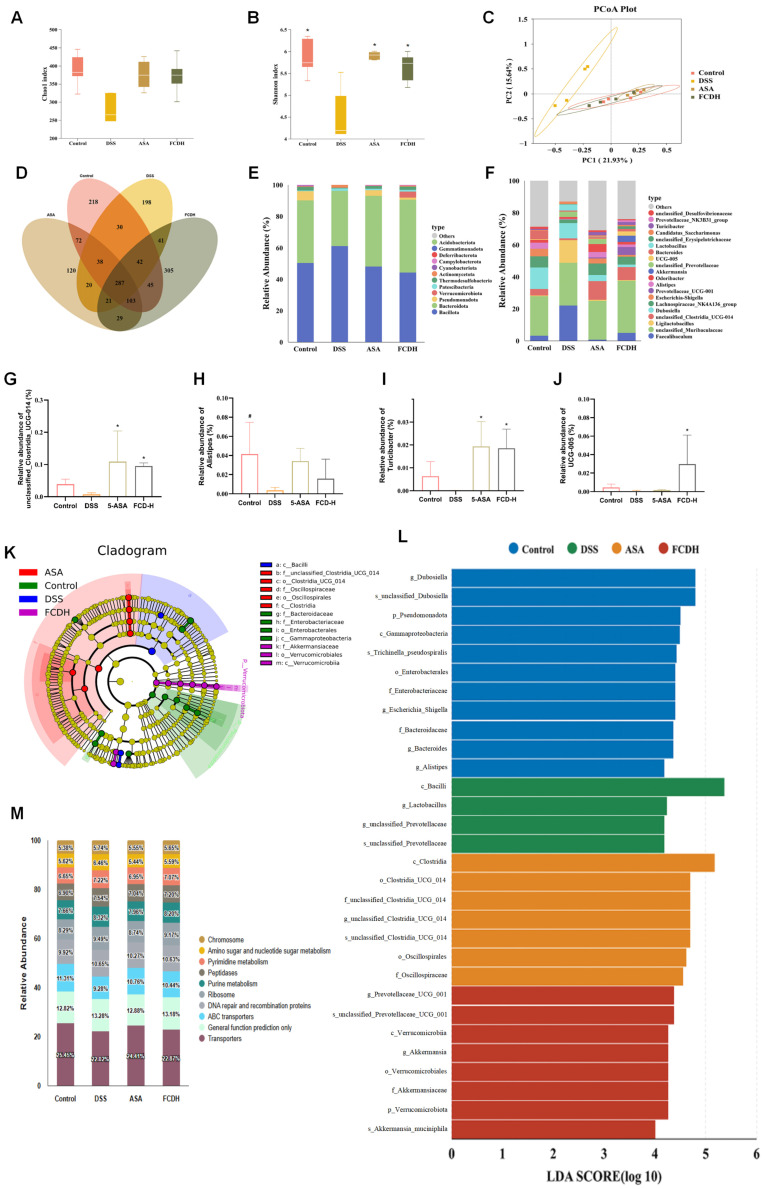
FCD ameliorated DSS-induced gut microbiota disorder. (**A**,**B**) α-diversity analysis was evaluated by Chao1 index and Shannon index. (**C**) Principal Co-ordinates Analysis (PCoA). (**D**) OUTs Venn diagram. (**E**,**F**) The composition of intestinal flora in phylum and genus level. (**G**–**J**) Differential flora at the genus level between groups; Data are presented by the mean (*n* = 5). (**K**) Cladogram based on LEfSe analysis. (**L**) Histograms of LDA scores. (**M**) Relative Abundance Map of Functional Genes. Compared with the CON group, # *p* < 0.05; compared with the DSS group, * *p* < 0.05.

Intestinal microbiota, essential for maintaining gut homeostasis, plays a pathogenic role in the initiation and progression of intestinal inflammation when dysbiosis occurs, Therefore, we used 16S rRNA gene sequencing to analyse the contents of the colon. The OTU counts for the four groups (Control, DSS, 5-ASA and FCD-H) were 218, 198, 120 and 305, respectively (Figure 6D). Evaluating intestinal flora α-diversity using the Chao1 and Shannon indices revealed that DSS-induced α-diversity declined, whereas FCD treatment reversed this trend (Figure 6A,B). PCoA and NMDS analyses further revealed differences in the overall microbial community structure among the Control, DSS, 5-ASA, and FCD-H groups (Figure 6C).

At the phylum level, *Bacillota* increased and *Bacteroidota* decreased in the DSS group compared to the control group. However, FCD and 5-ASA treatment reversed this trend, as demonstrated by reduced *Bacillota* and increased *Bacteroidota*. FCD significantly improved dysbiosis in *unclassified_Clostridia_UCG-014*, *UCG-005*, *Lactobacillus* and *Turicibacter* (Figure 6E–J). LEFSE analysis (LDA > 3.0) was used to investigate the presence of biomarkers in the Control, DSS, 5-ASA and FCD-H groups. At the genus level, significant differences in biomarkers were observed in the control group for the following genera: *Dubosiella, Alistipes, Bacteroides, Escherichia_Shigella* and *Dubosiella*. In the DSS group, significant differences in biomarkers were observed for *Lactobacillus and unclassified_Prevotellaceae*. Within the 5-ASA group, *unclassified_Clostridia_UCG_014* showed significant differences as a biomarker. In the FCD-H group, *Prevotellaceae_UCG-001 and Akkermansia* were identified as biomarkers. In conclusion, FCD was effective in alleviating the imbalance in intestinal flora associated with DSS (Figure 6K,L).

The relative abundance distribution of functional genes in the Control, DSS, ASA, and FCDH groups is as follows: Transporters were among the most abundant functional categories across all groups (accounting for 22.02–25.45%), while the abundance of metabolism-related functions (e.g., purine metabolism, pyrimidine metabolism) exhibited gradient changes among different treatment groups. The abundance of “only general functional predictions” in the DSS group (13.28%) was significantly higher than that in the control group (12.82%), while the abundance of ABC transporters in the FCDH group (10.44%) was slightly lower than that in the ASA group (10.76%) (Figure 6M). These intergroup differences in functional gene abundance indicate that different treatments exert specific regulatory effects on the functional gene composition of the samples.

## 4. Discussion

UC is a chronic, recurrent inflammatory disease characterised by common biological phenotypes, including diarrhoea, bloody stools, and weight loss, which significantly impacts patients’ quality of life. FCD is a traditional Xizang herbal medicine with potential immunomodulatory and anti-inflammatory effects. Nevertheless, its effects on UC remain unclear. The present study aims to investigate the protective effects of FCD and its underlying mechanisms in a DSS-induced UC mouse model.

In this study, UPLC–MS/MS was first employed for compositional analysis, ultimately identifying Sipeimine, Peiminine, Peimine, and Peimisine as the primary representative components likely playing a central role [30,31,32].This study established a UC mouse model using 3% DSS. The DSS group exhibited a range of adverse outcomes, including weight loss, shortened colons, increased spleen-to-body weight ratios, reduced food intake, and bloody stools. Detailed microscopic analysis of the tissue samples revealed the presence of mucosal erosion, accompanied by the destruction of both the submucosal and serosal layers. Conversely, the 5-ASA and FCD groups exhibited a marked reduction in DAI scores, indicating superior clinical and histological outcomes in comparison to the model group, accompanied by a significant amelioration of inflammatory symptoms. The mucus secreted by goblet cells forms a protective layer over the surface of gastrointestinal epithelial cells, thereby effectively isolating pathogenic microorganisms from invasion. Inflammation has been demonstrated to damage these goblet cells, resulting in reduced mucin secretion [33]. Using of AB-PAS staining facilitates the visualisation of acidic mucins in colon tissue, which manifest as blue, while neutral mucins are characterised by a purple appearance. The findings demonstrated a substantial decrease in mucin levels within the colon of the model group, while both the 5-ASA group and the FCD group exhibited a significant increase in mucin content. The results of this study indicate that FCD substantially alleviates DSS-induced UC-associated symptoms.

The oral administration of DSS has been demonstrated to induce severe damage to the colonic mucosa, characterised by increased intestinal permeability, mucosal thickening, and extensive inflammatory cell infiltration, ultimately leading to severe intestinal inflammation [34]. Research indicates that multiple pro-inflammatory cytokines (e.g., IL-1β, IL-6, IL-10, and TNF-α) play a pivotal role in the inflammatory response of UC and contribute to the onset and progression of the disease [35,36]. In the DSS-induced colitis model, elevated levels of inflammatory markers IL-1β, IL-6, IL-10, and TNF-α can be observed. The excessive secretion of these substances has been demonstrated to disrupt immune homeostasis, thereby exacerbating intestinal inflammation and mucosal ulceration. ELISA results indicate that, in comparison with the control group, the DSS group demonstrated increased inflammatory cell infiltration and elevated inflammatory cytokines, whereas both the 5-ASA group and the FCD group exhibited notable improvements.

In the absence of underlying pathologies, the gastrointestinal tract maintains an intact barrier function that prevents the invasion of pathogens, regulates the immune system, and maintains normal bodily functions [37,38]. The intestinal barrier primarily consists of the mucus barrier and the mechanical barrier [39,40]. The superficial layer of the mucosa is composed of epithelial tissue, forming a crucial physiological boundary between the internal environment and the external world. The function of these epithelial cells is to separate the contents of the cavity from the external environment, and it has been determined that tight junction proteins are key components in this process of epithelial recognition [41,42]. Disruption of the epithelial TJ barrier in the gut allows the entry of harmful molecules into the lumen, resulting in the disruption of the mucosal immune system and subsequent inflammation [43]. In the typical instance of UC, there is a downregulation of the three tight junction proteins ZO-1, Occludin and Claudin-1, which leads to a disruption of the intestinal barrier function [44,45,46,47]. Therefore, the present study sought to assess colonic intestinal integrity by examining the distribution and expression of tight junction proteins in the colon. The experimental findings demonstrated that the administration of FCD significantly ameliorated the impairment of the intestinal barrier induced by UC. Patients suffering from UC frequently exhibit signs of gut microbiota dysbiosis, which is primarily characterised by a decrease in microbial diversity [48]. Extensive research indicates that DSS induces changes in the microbial diversity and community composition of mice. A substantial body of research has demonstrated that the administration of faecal microbiota transplantation (FMT) from healthy human donors into DSS-induced UC mouse models can effectively alleviate the associated symptoms in these animals [49]. Consequently, the restoration of gut microbiota homeostasis has become a key focus of current UC research [50,51,52]. This study used 16S rRNA sequencing analysis to reveal that FCD can reverse DSS-induced gut dysbiosis and enhance the diversity of the gut microbiota. It can also modulate the abundance of specific bacterial genera. An increase in Bacteroidetes and a decrease in Bacillota were observed, consistent with previous reports [53]. Additionally, the abundances of *unclassified_Clostridia_UCG-014*, *UCG-005* and *Turicibacter* increased significantly within the FCD-H group.

In this experiment, the relative abundance of the purine metabolism pathway in the gut microbiota of mice in the Control group was 7.68%. while in DSS-induced UC model mice, this pathway’s abundance significantly increased to 9.32%. This aligns with previous findings that “the gut microbiota functional pathways in UC model mice involve purine metabolism”, suggesting that abnormal activation of the purine metabolism pathway is one characteristic of UC gut microbiota dysregulation and may contribute to the inflammatory pathological process. Following administration of ASA and FCDH, pathway abundance decreased to 7.96% and 8.20%, respectively, approaching baseline levels in the Control group. This aligns with the regulatory trend reported in the literature that “Luteolin treatment for UC reduces gut microbiota-associated purine metabolic activity” [54]. This suggests that ASA and FCDH may target the purine metabolism pathway by inhibiting its excessive activation, reshaping the gut microbiota metabolic functional network, and reducing inflammation-related metabolites, thereby alleviating UC-associated intestinal inflammatory damage. This provides metabolic pathway-level evidence for subsequent analysis of their specific action targets. In this experiment, the relative abundance of ABC transporters in DSS-induced UC model mice (9.28%) was significantly lower than that in the blank control group (11.31%). consistent with literature reports indicating a trend toward downregulation of certain ABC transporter subtypes in the colon of patients with active UC. This confirms that abnormal transporter abundance represents a common pathological alteration associated with UC. Following administration of ASA and FCDH, the relative abundance of these transporters recovered to 10.76% and 10.44%, respectively, approaching control levels. echoing literature findings that UC therapeutics can modulate ABC transporter expression. Combined with reports indicating that altered expression of this transporter is a functional contributor to UC pathogenesis rather than merely a concomitant feature of epithelial injury, these results provide experimental support for ASA and FCDH intervention in UC through regulating ABC transporter abundance. They also substantiate the potential therapeutic value of this transporter as a target for UC treatment [55].

## 5. Conclusions

In conclusion, FCD not only protects the integrity of the intestinal barrier by enhancing the distribution and expression of tight junction proteins, but also ameliorates DSS-induced UC symptoms by improving dysbiosis in the gut microbiota of mice. This study provides a theoretical foundation and basis for the further drug development and clinical application of FCD, while also opening new avenues for the prevention and treatment of ulcerative colitis.

## Figures and Tables

**Table 1:** Primer sequences used for RT-qPCR.

Gene Name	Primers	Sequence (5′-3′)
GAPDH	Forward	TGTGTCCGTCGTGGATCTGA
	Reverse	TTGCTGTTGAAGTCGCAGGAG
ZO-1	Forward	GCCGCTAAGAGCACAGCAA
	Reverse	TCCCCACTCTGAAAATGAGGA
Occludin	Forward	TTGAAAGTCCACCTCCTTACAGA
	Reverse	CCGGATAAAAAGAGTACGCTGG
Claudin-1	Forward	GGGGACAACATCGTGACCG
	Reverse	AGGAGTCGAAGACTTTGCACT

**Table 2:** The composition of FCD medicated serum was identified based on UPLC-MS/MS.

No.	MS2 Name	Rt (min)	Adduct	mz	Precursor MZ	Mz_Error	Formula	MS2
1	Isonicotinic acid	0.81	[M+H]+	124.0388	124.0393	−4.2	C_6_H_5_NO_2_	124; 123; 95
2	Paeonol	1.70	[M-H_2_O+H]+	149.0592	149.0597	−3.3	C_9_H_10_O_3_	103.1; 107; 131
3	Gramine	3.48	[M+H]+	175.1225	175.1230	−2.7	C_11_H_14_N_2_	175.1; 148.1; 158.1
4	*N*-Feruloylputrescine	3.60	[M+H]+	265.1538	265.1547	−3.5	C_14_H_20_N_2_O_3_	177.1; 265.2; 248.1
5	Sipeimine	4.06	[M-H_2_O+H]+	412.3195	412.3210	−3.7	C_27_H_43_NO_3_	412.3
6	Peiminine	4.30	[M-H_2_O+H]+	412.3198	412.3210	−3	C_27_H_43_NO_3_	412.3
7	Peimine	4.31	[M-H_2_O+H]+	414.3363	414.3366	−0.7	C_27_H_45_NO_3_	414.3; 70.1; 86.1
8	Jervine	4.49	[M+H]+	426.2991	426.3003	−2.7	C_27_H_39_NO_3_	426.3; 311.2; 84.1
9	Peimisine	4.67	[M+H]+	428.3145	428.3159	−3.4	C_27_H_41_NO_3_	428.3; 114.1; 410.3
10	Solanidine	5.46	[M+H]+	398.3407	398.3418	−2.8	C_27_H_43_NO	398.3; 163
11	Quinic acid	0.90	[M-H_2_O-H]−	173.0453	173.0455	−1.2	C_7_H_12_O_6_	129.1; 173; 57
12	1-Kestose	1.52	[M-H]−	503.1619	503.1617	0.3	C_18_H_32_O_16_	89; 341.1; 161
13	2-Isopropylmalic acid	3.65	[M-H]−	175.061	175.0612	−1.1	C_7_H_12_O_5_	115; 175.1; 113.1
14	Quercetin 3-gentiobioside	4.66	[M-H]−	625.1407	625.1410	−0.5	C_27_H_30_O_17_	300; 625.1; 301
15	Taxifolin	4.92	[M-H]−	303.0509	303.0510	−0.5	C_15_H_12_O_7_	125; 285; 177
16	Azaleatin	6.00	[M-H]−	315.0508	315.0510	−0.6	C_16_H_12_O_7_	315.1; 300; 301

## Data Availability

The original contributions of this study are documented in the article/Supplementary Materials. Further enquiries may be directed to the corresponding author.

## Supplementary Materials

The following supporting information can be downloaded at: https://www.mdpi.com/article/10.3390/nu18060970/s1, Figure S1: Liver tissue H&E staining.

## Abbreviations

The following abbreviations are used in this manuscript:

UC	Ulcerative Colitis
DSS	Dextran Sulfate Sodium
FCD	Tibetan *Fritillaria cirrhosa* D. Don extract
ZO-1	Zonula occludens-1
IBD	Inflammatory bowel disease
TJ	Tight Junction
5-ASA	5-Aminosalicylic Acid
NSAIDs	Non-steroidal anti-inflammatory drugs
GAPDH	Glyceraldehyde-3-Phosphate Dehydrogenase
UPLC-MS/MS	Ultra Performance Liquid Chromatography—Tandem Mass Spectrometry
LC	Liquid Chromatography
H&E	Haematoxylin and eosin
AB-PAS	Alcian Blue—Periodic Acid Schiff
DAB	3,3′-Diaminobenzidine
BCA	Bicinchoninic Acid Assay
ANOVA	Analysis of Variance
PCoA	Principal Co-ordinates Analysis
NMDS	Non-metric Multidimensional Scaling
LEFSE	Linear Discriminant Analysis Effect Size
