# Supplementary figures and images for: Tibetan Fritillaria cirrhosa D. Don Extract Ameliorates DSS-Induced Ulcerative Colitis by Repairing Damage to the Intestinal Mucosal Barrier and Regulating the Gut Microbiota
Source: Nutrients. 2026 Mar 19;18(6):970. doi: 10.3390/nu18060970 (PMC13028620; doi:10.3390/nu18060970)

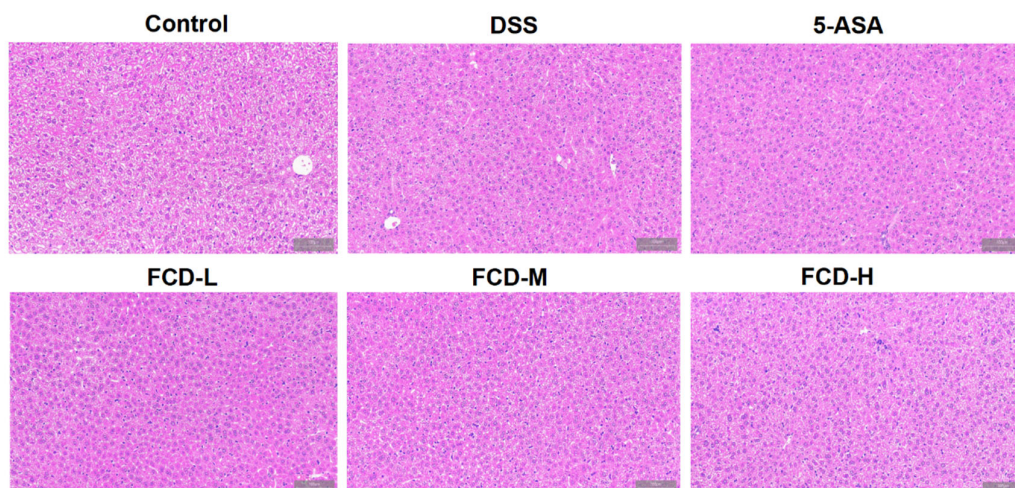

Figure S1. Liver tissue H&E staining.

Supplement: Supplementary file 1 [file nutrients-18-00970-s001.zip › nutrients-4049534-supplementary.pdf]
